# IMP3 can predict aggressive behaviour of lung adenocarcinoma

**DOI:** 10.1186/1746-1596-7-165

**Published:** 2012-11-28

**Authors:** Renata Beljan Perak, Merica Glavina Durdov, Vesna Capkun, Veljka Ivcevic, Antonia Pavlovic, Violeta Soljic, Mari Peric

**Affiliations:** 1 Institute for pathology, forensic medicine and cytology Clinical Hospital Center Split, Split, Croatia; 2 Department for nuclear medicine, Clinical Hospital Center Split, Split, Croatia; 3 Pulmology Department Clinical Hospital Center Split, Split, Croatia; 4 Institute for pathology, forensic medicine and cytology, University Hospital Mostar, Mostar, Bosnia and Herzegovina; 5 Institute for radiology Clinical Hospital Center Split, Split, Croatia

**Keywords:** Lung adenocarcinoma, Aggressive phenotype, TTF-1, Napsin A, IMP-3

## Abstract

**Background:**

Lung cancer most often presents as an inoperable tumour and the diagnosis is usually performed on a small biopsy/cytology specimen. In the group of non small cell lung cancer - not otherwise specified, adenocarcinoma phenotype can be determined immunohistochemically using TTF-1 and Napsin A. Expression of oncofetal protein IMP3 in human cancer is associated with poor differentiation and aggressive behaviour. In the present study expression of IMP3 was correlated with expression of TTF-1 and Napsin A, histological subtype and clinical stage of lung adenocarcinoma. We were interested whether distant metastases are associated with IMP3 overexpression, regardless of the histologic subtype of adenocarcinoma.

**Methods:**

In retrospective study, consecutive series of 105 patients with advanced lung adenocarcinoma diagnosed from 2006 to 2009 in Clinical Hospital Center Split, Croatia, were analysed. Clinical data were collected from the Pulmology Department and time of death from the Mortality Registry. Paraffin blocks of bronchoscopic biopsies were collected from the Institute of Pathology and 15 cases excluded from the analysis due to insufficient material. Expression of IMP3, Napsin A and TTF-1 were analysed by indirect enzyme immunohistochemistry. Statistical analysis was performed and *P* values less than 0.05 considered significant.

**Results:**

Of 90 patients, 71 (78%) were males and 19 (22%) females. Median age for males was 61.5 years (min-max 43–83) and for females 61 years (min-max 44–86). Pleural effusion was found in 15 (16.6%) and distant metastases in 45 (50%) cases. According to histological subtypes, there were 34 acinar, 2 lepidic, 2 papillary and 52 solid subtypes. IMP3 overexpression was found in 63 cases (70%) and was correlated with solid subtype (*P* = 0.002) and negative/weak Napsin A expression (*P* = 0.004). Strong Napsin A expression correlated with TTF-1 expression (*P* = 0.003) and lower histological grades (*P* = 0.031). Patients with IMP3 overexpression more often had distant metastases than patients with negative IMP3, 55.5% versus 33.3% (*P* = 0.033). Non solid subtypes with IMP3 overexpression developed distant metastasis more common than non solid subtypes with negative IMP3, 72% versus 35% (*P* = 0.028).

**Conclusions:**

Expression of IMP3 correlates with solid subtype and with distant metastases regardless of histological subtype of lung adenocarcinoma.

**Virtual slides:**

http://www.diagnosticpathology.diagnomx.eu/vs/1966211581795258

**Zusammenfassung:**

**Hintergrund:**

Das Lungenkarzinom kommt meistens als nicht resektabler Tumor vor und die Diagnose kann nur in kleinen Biopsaten oder zytologisch gestellt werden. In der Gruppe der nicht kleinzelligen Lungenkarzinome kann der nicht anders spezifizierte Adenokarzinom Phänotyp mit Hilfe der Antikörper TTF-1 und Napsin A diagnostiziert werden. Die Expression des onkoföetalen Proteins IMP3 ist bei humanen Karzinomen mit agressivem Verhalten und metastatischem Potential verbunden. In dieser Studie korreliert die Expression von IMP3 mit TTF-1, Napsin A, histologischem Typ und klinischem Staging des Lungenkarzinoms. Wir waren daran interessiert, ob Fernmetastasen mit IMP3 Überexpression assoziiert sind, unabhängig von der histologischen Subtyp von Adenokarzinom.

**Methode:**

In der retrospektiven Studie wurden die von 2006 bis 2009 im Klinischem Krankenhaus Split, Kroatien diagnostizerte Adenokarzinome der Lunge von 105 Patienten analysiert. Die klinischen Daten stammten aus der Abteilung für Pulmologie und im Falle des Todes vom Todesregister. Die Paraffinblöcke der primären Lungenbiopsate dieser Patienten wurden im Institut für Pathologie mit der indirekter Enzym - Immunohistochemie mittels Kombination der Antikörper gegen IMP3, Napsin A und TTF1 untersucht. 15 Fälle aus der Analyse aufgrund unzureichender Material ausgeschlossen. Es wurde eine statistische Untersuchung durchgeführt und Werte weniger als 0.05 *P* wurden als statistisch signifikant bezeichnet.

**Ergebnisse:**

Von 90 Patienten mit Lungencarcinom waren 71 (78%) mänlich, durchschnittliches Alter war für Männer 61.5 Jahre (min-max 43–83) und 61 Jahre für Frauen (min-max 44–86). Pleurale Effusionen fand man in 15 Fällen (16.6%) und Fernmetastasen in 45 (50%) Fällen. Histologische Sybtypen waren: 2 lepidic Karzinome, 34 azinäre Karzinome, 2 papilläre und 52 solide Karzinome. IMP3 war exprimiert in 63 Fälle (70%). Positive IMP3 Expression war mit solidem Typ (*P* = 0.002) und negativer Napsin A Expression (*P* = 0.004) assoziert. Napsin A Expression war mit niedrigem Gradus (P = 0.031) und positiver TTF-1 Expression (*P* = 0.003) assoziert. Patienten mit IMP3 Überexpression öfter hatten Fernmetastasen als Patienten mit negativen IMP3, 55.5% versus 33.3% (*P* = 0.033). Non solide Subtyp mit IMP3 Überexpression entwickelten Fernmetastasen Meer häufiger als nicht festem Subtyp mit negativen IMP3, 72% versus 35% (*P* = 0.028).

**Schlussworte:**

Die Expression von IMP3 ist mit negaativer Expression von Napsin A, solidem Subtyp und Metastasen verbunden und hat praktische predictive Werte in der pathologischen Diagnose des Adenokarzinoms der Lunge. Die Expression von IMP3 korreliert mit soliden Subtyp und mit Fernmetastasen unabhängig von histologische Subtyp Lungenadenokarzinom.

## Background

Lung cancer is the leading cause of cancer mortality in the world, with the highest incidence in the Western World and poor overall survival [1]. It presents a complex disease with different phenotypes, variabile responses to therapy and probably different relationships to specific carcinogens [2]. Small cell lung carcinomas (SCLC) and non-small cell lung carcinomas (NSCLC) are wide and clinically relevant groups of lung cancer. The most common histologic type of NSCLC is adenocarcinoma (ADC), accounting for nearly half of all lung cancers [3]. There is general agreement that squamous cell carcinoma (SCC) and SCLC have the highest accuracy of diagnosis in small preoperative specimens, whereas ADC is less accurately diagnosed due to its histological complexity [4]. Due to advanced clinical stage at presentation, up to 90% of diagnoses are established on small biopsy or cytology specimen [5], but because of the paucity of bronchoscopic material, careful selection of ancillary methods (like histochemical mucin stain, immunohistochemistry and polymerase chain reaction) should be done [6]. Multiplex panel dual immunohistochemistry using p63/CK 5 and TTF-1/Napsin A are useful in subcategorising NSCLC-NOS into SCC and ADC phenotypes [7]. Combination of TTF-1 and Napsin A highly improved sensitivity and specificity for diagnosing lung adenocarcinoma [8]. TTF-1 is a nuclear tissue-specific DNA-binding protein mainly expressed in thyroid follicular cells, type II pneumocytes and nonciliated bronchiolar epithelial cells. Its function in lungs is transcriptional activation of surfactant proteins and secretory proteins of Clara cells [9]. Napsin A is aspartic proteinase involved in the maturation of the surfactant B. It is expressed in the cytoplasm of type II pneumocytes and Clara cells, in proximal tubular renal epithelium and exocrine pancreas [10,11]. In the last few years IMP3, member of insulin-like growth factor II mRNA binding protein family, was investigated in different malignant neoplasms because its overexpression is generally associated with aggressive and advanced tumours [12]. Normally, IMP3 is expressed in developing tissues during embryogenesis and plays the role in RNA trafficking and stabilisation, cell growth and migration [13]. In adult tissues IMP3 expression is low or undetectable, but in malignant tumors is strongly expressed [14,15]. In most studies increased expression of IMP3 correlates with aggressive biological behaviour of the tumour [16,17]. Expression of IMP3 in series of lung cancer was analysed in few studies. According to these articles, IMP3 overexpression in high grade neuroendocrine carcinoma [18], non small cell lung carcinoma [19] and adenocarcinoma [20] correlated with poor differentiation and advanced stage of disease. In this study we analysed expression of IMP3 in histological subtypes of lung adenocarcinoma and its impact on clinical staging.

## Methods

**Figure 1:**
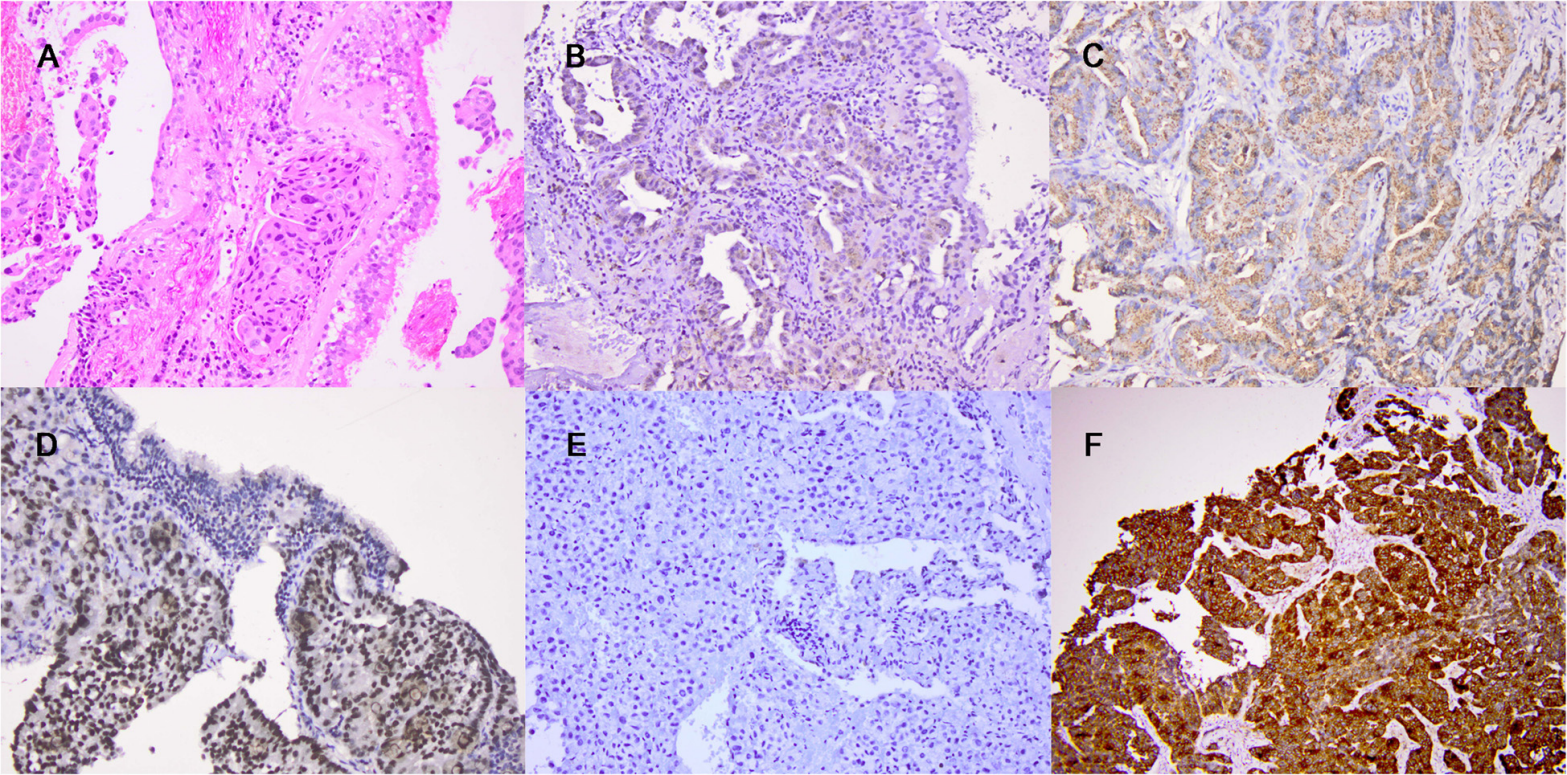
**Expression of TTF-1, Napsin A and IMP3 in lung adenocarcinoma.A** Tumor tissue infiltrates bronchal mucosa, **B** Nuclei of malignant cells are positive to TTF-1, **C** Weak cytoplasmatic granular positivity to Napsin A. **D** Strong granular cytoplasmatic positivity to Napsin A. **E** Negative expression od IMP3, **F** positive expression of IMP3 (200x).

From January 2006 till December 2009, 807 new cases of lung cancer were diagnosed at the Institute of pathology, forensic medicine and cytology, Clinical Hospital Center Split, Croatia. The consecutive series of 105 patients with primary inoperable adenocarcinoma were investigated. Clinical data (age, sex, tumor size, clinical staging) were collected from the hospital records and time of death from the Mortality Registry. Overall survival was evaluated for outcome analysis. Paraffin blocks were collected from the Institute of pathology, forensic medicine and cytology and 15 cases were excluded due to insufficient material. Indirect immunohistochemical analysis was performed on 5-μm sections using primary monoclonal mouse antibodies against IMP3 (dilution 1:200), Napsin A (dilution 1:200) and TTF-1 (dilution 1:100), EnVision/HRP and chromogen 3,3'-diaminobenzidine (all reagents DAKO, Glostrup, Denmark). The slides were analysed with light microscope Olympus 51BX by two pathologists. Diffuse cytoplasmic staining for IMP3, nuclear staining for TTF-1 and granular cytoplasmic staining for Napsin A were considered positive. Expression of TTF-1 and IMP3 was assessed as positive or negative and expression of Napsin A as negative/weak or strongly positive (Figure 1). Cases with more than 10% of positive cells were considered as positive. Statistical analysis was performed using the SPSS 19 system for Windows and *P* value less than 0.05 was considered statistically significant.

## Results

In the sample were 90 patients, 71 (78%) males and 19 (22%) females. Median age for males was 61.5 years (min-max 43–83) and for females 61 years (min-max 44–86). In five years of follow up, 79 (87%) patients died. The mean overall survival was 8 months (95% CI 1–34 months). Median survival was 2,5 times longer in patients without distant metastases: 9 months (SE 1.8 months) (95% CI 5.4-12.6) compared to patients with metastases: 4 months (SE 1.1) (95% CI 1.9-6.1) (log rank 6.5 P = 0.011). The pleural effusion was present in 15 (16.6%) cases and in 14 patients was cytologically malignant. In the beginning of diagnostic procedure 19 patients were examined with multislice computed tomography of the thorax and upper abdomen and staged into T2 (5), T3 (7) and T4 (7) categories; the remaining patients only had chest radiography. Median tumour size was 58.5 mm (min-max 21–112). Radiographic evidence of significantly enlarged and presumably positive regional lymph nodes had 51 (56.6%) patients. In the analysed period 45 (50%) patients developed distant metastases in bones (14), brain (10), liver (5), adrenal glands (4), or multiple sites (12). Histological grading was performed according to the presence of solid component and cytological atypia [21], resulting in 18 well differentiated, 23 moderately differentiated and 49 poorly differentiated tumours. According to the criteria set forth by the WHO (2004), there were 34 acinar, 2 lepidic, 2 papillary and 52 solid subtypes; in further analysis solid subtype has been compared to others.

According to Table 1, positive expression of IMP3 was found in 61 (67.7%) cases. Median age of patients with positive IMP3 expression was 58.5 years (min-max 43–83) and of patients with negative IMP3 expression 63 years (53–79) (Z = 1,75 *P* = 0.08). Median tumor size was 58.5 mm (min-max 21–112) in IMP3 positive patients and 60 mm (min-max 22–108) in IMP3 negative patients (Z = 0.82 *P =* 0.411). No statistically significant correlations were observed with sex (*χ*^2^ =0156 *P* = 0.693), pleural effusion (*χ*^2^ = 3.5 *P* = 0.063), histological grade (*χ*^2^ =2.499 *P* = 0.287) and TTF-1 expression (*χ*^2^ =0.276 *P* = 0.599). Overexpression of IMP3 correlates to solid subtype (*χ*^2^ =9.4 *P* = 0.002) and negative Napsin A expression (*χ*^2^ =8.25 *P* = 0.004). In the group of 52 cases with solid subtype, 43 (82.6%) had IMP3 overexpression and in the group of 38 cases with other subtypes 20 (52.6%). IMP3 overexpression correlated with presence of distal metastases (staging M1b) (*χ*^2^ = 6.844 *P* = 0.033).

**Table 1:** Expression of IMP3 in patients with lung adenocarcinoma

**Variable**		**N**	**IMP3 (N = 90)**		***P***
			**Negative N (%)**	**Positive N (%)**	
Gradus	1	18	8 (29.6)	10 (15)	0.287
	2	23	7 (25.9)	16 (25)	
	3	49	12 (44)	37 (58)	
Subtype	solid	52	9 (33)	43 (68)	0.002
	others	38	18 (66)	20 (31)	
Napsin A	negative/light	60	12 (46)	48 (77)	0.004
	strong positive	28	14 (53)	14 (22)	
TTF-1	negative	31	9 (33.3)	22 (39.2)	0.599
	positive	52	18 (66.6)	34 (60.7)	
Pleural effusion	no	29	6 (42.8)	3 (76.6)	0.063
	yes	15	8 (16.6)	7 (23.3)	
T staging	2	10	4 (33.3)	6 (21.4)	0.458
	3	10	2 (16.6)	8 (28.5)	
	4	20	6 (50)	14 (50)	
N staging	1	20	9 (64)	11 (52.3)	0.612
	2	14	5 (35)	9 (42.8)	
	3	1	0	1 (4)	
M staging	0	31	10 (37)	22 (34.9)	0.033
	1a	14	8 (29.6)	6 (9.5)	
	1b	45	9 (33.3)	36 (55.5)	

*χ*^2^ test.

Table 2 shows the distribution of metastases according to IMP3 overexpression in patients with non solid subtype of adenocarcinoma. Among 18 patients with distant metastases, 13 (72%) were IMP3 positive. Among 20 patients without distant metastases, IMP3 was positive in 7 (35%) cases. In non solid subtypes, IMP3 overexpression was more often found in patients who developed metastasis (Fisher exact test *P* = 0.028). IMP3 positive cases had shorter time of overall survival, but result is not statistically significant (log rank 0.14 *P* = 0.713). Patients with negative IMP3 expression had mean survival 9 months (SE 2 months) (95% CI 5–12 months) and patients with positive IMP3 had mean survival 9 months (SE 1 month) (95% CI 4–6 months).

**Table 2:** IMP3 in non solid subtypes of lung adenocarcinoma and distant metastasis

**Non solid subtypes (N = 38)**	**IMP3**		**Distal metastasis**	***P***
	**negative**	**positive**		
18	5 (27.7)	13 (72.2)	yes	0.028
20	13 (65)	7 (35)	no	

Fisher exact test.

Expression of Napsin A was not related to sex (*χ*^2^ =1.577 *P* = 0.209) and pleural effusion (*χ*^2^ = 0 *P* = 1). In cases with negative expression of Napsin A median tumor size was 59 mm (min-max 23–112) and with positive Napsin A expression 59.98 mm (min-max 21–108) (Z = 0.518 *P = *0.605). Napsin A expression correlated with TTF-1 positivity (*χ*^2^ = 8.986 *P* = 0.003) and lower histological grade (*χ*^2^ = 8.986 *P* = 0.003).

## Discussion

About 70-80% of lung carcinomas fall under the classification of NSCLC with adenocarcinoma as the most common subtype [22]. In the past, all subtypes of NSCLC received the same therapy and subclassification was not important for the treatment or prognosis. Recent development in the treatment strategies necessitates further cytological and histological subtyping of NSCLC. Promotor methylation was more common find in squamous carcinoma and correlated to pleural indentation [23]. Activated gene mutations are more common found in adenocarcinoma [2]. New biologically targeted chemotherapies, such as bevacizumab, have clinical benefits in patients with ADC, but strong contraindications in SCC cases. There are therapies that target EGFR mutations and ALK fusion genes which are found almost exclusively in adenocarcinomas. Architecture of pulmonary adenocarcinoma is very heterogeneous. Morphologic features, as specified in the WHO classification 2004, have been the standard for NSCLC subtyping, but according to Terry et al. [7], 25% of bronchoscopic biopsies cannot be subclassified by morphology alone so immunohistochemical and/or genetic analyses are necessary. Immunohistochemical markers TTF-1 and Napsin A are nowadays a useful tool in adenocarcinoma identification and their dual use improves diagnostic accuracy, but does not give us data about prognosis of cancer which often has high metastatic potential. There have been some studies about prognostic value of some histological characteristics [9,21] or methylation markers [23]. We tried to obtain this important prognostic information using antibody to oncofetal protein IMP3, previously identified as a marker of aggressive behaviour in various tumours.

Expression of IMP3 was analysed in relation with histological grade proposed by Barletta et al. [21], but no significant correlation was found (*P* = 0.287). According to some authors, [24] there is no well-established histological or cytological grading scheme for NSCLC and currently applied subjective grading is not prognostically relevant. In our series solid subtype was the most common and it was analysed in relation to other subtypes (acinar, lepidic and papillary) grouped together. One case of papillary subtype had characteristics of recently described stromal micropapillary pattern [25,26]. IMP3 overexpression correlated with solid subtype (*P* = 0.002). In the series of 89 surgically resected specimens Findeis-Hosey et al. have found that positive IMP3 expression was strongly correlated with poorly differentiated adenocarcinoma and solid component of myxed subtype adenocarcinoma [20]. In our study, expression of TTF-1 and Napsin A were 61% and 51%, respectively. TTF-1 expression value was within reported range [9], but percentage of Napsin A positive cases was surprisingly low [27]. Possible explanation for this could be high proportion of solid subtype in our series, although technical problems in sampling and fixation could not be excluded. Expression of Napsin negatively correlated with poor differentiation and 40 (76.9%) cases of histologic grade 3 were Napsin A negative (*P* = 0.031). Expression of TTF-1 did not correlate with grade (*P* = 0.542), which confirmed objection of Ueno et al. [27] that TTF-1 showed no association with the grade of tumour differentiation. Both TTF-1 and Napsin A were negative in 28 cases, majority of those were of solid subtype and immunohistochemically negative to Cytokeratin 5/6. The proportion of dual negative cases in our study was higher than in study of Turner et al. [8], 32% versus 10.6%, respectively which could be explained by high percentage of solid subtype in our sample. IMP3 overexpression correlated with negative expression of Napsin A (*P* = 0.004), but correlation of IMP3 and TTF-1 was not found (*P* = 0.599).

During five-year follow-up, out of 90 patients with inoperable lung adenocarcinoma, 79 (8.77%) died. The mean overall survival was 8 months (95% CI 1–34 months). IMP3 overexpression had no influence on mean overall survival (*P* = 0.712). We did not find statistically significant difference in tumour size (*P* = 0.411), T staging (*P* = 0.458) or N staging *(P* = 0.612) among IMP3 overexpressed and IMP3 negative cases. Findeis-Hosey et al. found that IMP3 is more frequently expressed in larger tumours with positive lymph nodes or higher clinical stage, but the difference was not statistically significant [20].

In our series, 45 patients developed distant metastasis to the brain, liver, adrenal glands, bones or to multiple sites. Quite often symptoms of metastases were the first sign of the malignancy. IMP3 overexpression showed correlation with distant metastases regardless of histological subtype (*P* = 0.033). Further series are needed to confirm these results and eventually establish IMP3 in initial immunohistochemical panel for small bronchoscopic biopsy/cytology, as a marker for aggressive behaviour and higher metastatic potential of lung adenocarcinoma.

In conclusion, IMP3 overexpression correlated with solid subtype and negative expression of Napsin A, as well as distant metastasis regardless of hystological subtype. Our results confirm that positive IMP 3 expression predicts aggressive tumour behaviour in lung adenocarcinoma and can be used as a significant predictor of unfavorable outcome.

## Competing interests

The authors declare that they have no competing interests.

## Authors' contributions

RBP and MGD designed the study, analysed histological slides, interpreted the results and drafted the manuscript, AP, VI and MP collected pathological, clinical and radiological data, VC performed the statistical analysis and VS critically revised manuscript. All authors read and approved the final manuscript.
